# p21-activated kinase 1 (PAK1) expression correlates with prognosis in solid tumors: A systematic review and meta-analysis

**DOI:** 10.18632/oncotarget.8320

**Published:** 2016-03-24

**Authors:** Fang Fang, Jian Pan, Yi-Ping Li, Gang Li, Li-Xiao Xu, Guang-Hao Su, Zhi-Heng Li, Xing Feng, Jian Wang

**Affiliations:** ^1^ Institute of Pediatric Research, Children's Hospital of Soochow University, Suzhou 215025, Jiangsu, China

**Keywords:** PAK1, solid tumor, prognosis, survival, meta-analysis

## Abstract

p21 protein (Cdc42/Rac)-activated kinase 1 (PAK1) expression appears to be predictive of prognosis in various solid tumors, though the evidence is not yet conclusive. We therefore performed a meta-analysis to explore the relationship between PAK1 and prognosis in patients with solid tumors. Relevant publications were searched in several widely used databases, and 15 studies (3068 patients) were included in the meta-analysis. Pooled hazard ratios (HRs) and 95% confidence intervals (CIs) were calculated to evaluate the strength of the association between PAK1 and prognosis. Associations between PAK1 expression and prognosis were observed for overall survival (HR = 2.81, 95% CI = 1.07-7.39) and disease-specific survival (HR = 2.15, 95% CI = 1.47-3.16). No such association was detected for time to tumor progression (HR = 1.78, 95% CI = 0.99-3.21).Our meta-analysis thus indicates that PAK1 expression may be a predictive marker of overall survival and disease-specific survival in patients with solid tumors.

## INTRODUCTION

p21 protein (Cdc42/Rac)-activated kinase 1 (PAK1) is a member of the PAK family of proteins, which are effectors of small Rho GTPases (Cdc42 and Rac1) [1, 2]. PAK1 is involved in a variety of cellular functions, including cell motility, survival, mitosis, cytoskeletal rearrangement and angiogenesis [3]. In addition, PAK1 plays key roles in nuclear signaling and activation of the JNK/SAPK and p38MAPK pathways [4, 5]. Although it has been suggested that PAK1 influences the prognosis of various cancer types [3, 6–21], current knowledge of the contribution of PAK1 to cancer prognosis remains limited.

In the present study, we used a statistical approach to systematically investigate the association between PAK1 and the prognosis of solid tumors. Over the past decade, a series of studies have focused on the relationship between PAK1 expression and solid cancer prognosis [3, 6–21], but the results of those individual studies were not conclusive. We therefore performed a meta-analysis using a relatively large sample from 15 studies (3068 patients) with the aim of conclusively determining the relationship between PAK1 and prognosis in patients with solid tumors.

## RESULTS

### Studies and data included in this meta-analysis

**Figure 1:**
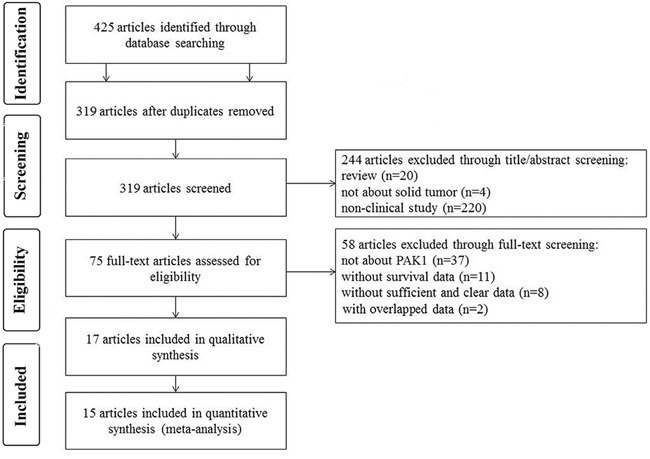
Flow chart of the study selection

Through searching and selection, a final list of 17 studies [3, 6–21] was collected for qualitative synthesis (Figure 1). The participants in the studies spanned different ethnicities (11 studies of Asians and 6 studies of Caucasians) and cancer types (3 studies of breast cancer, 2 colorectal cancer, 2 gastric cancer, 2 head and neck cancer, 2 ovarian cancer, 1 gastroesophageal junction adenocarcinoma, 1 glioblastoma, 1 hepatocellular carcinoma, 1 pancreatic cancer, 1 renal cell carcinoma, and 1 urothelial carcinoma of the upper urinary tract). Detailed information on these studies is summarized in Table 1. The studies from Aoki et al. and Zhu et al. investigated the prognostic utility of p-PAK1 only, and were not included in the quantitative synthesis (meta-analysis). Of the remaining 15 studies, 5 focused on overall survival (OS), 2 focused on disease-specific survival (DSS), 2 focused on disease-free survival (DFS), 1 focused on progression-free survival (PFS), 1 focused on recurrence-free survival (RFS), and the remaining 4 investigated more than one type of outcome endpoints. In total, the 15 studies eligible for meta-analysis provided a sample of 3068 patients with which to assess the relationship between PAK1 expression and solid tumor prognosis.

**Table 1:** Studies and data included in this meta-analysis

Author	Year	Patients' country of origin	Cancer type	No. of patients	Stage/Grade	Detection method	Percentage of high PAK1 expression, cutoff value	Median follow-up months	Outcome	Outcome definition	Survival analysis method
Holm	2006	Sweden	Breast cancer	284	Grade I-III	IHC	NA, groups3-5	166.8	RFS	surgery ∼ recurrence/breast cancer death	M
Aoki^a^	2007	USA	Glioblastoma	136	Grade 4	IHC	NA, NA	13.5	OS	surgery ∼ NA	M
Davidson	2008	Norway	Ovarian carcinoma	83	I-IV	IHC	57/83 (68.7%), >25% of cells	NA	PFS,OS	diagnosis∼recurrence, diagnosis ∼ death/last follow-up	KM
Liu	2009	China	Gastric cancer	40	I-IV	Western blotting	20/40 (50.0%), >1.43-fold	NA	DSS	NA	KM
Bostner	2010	Sweden	Breast cancer	786	NA	IHC	453/786 (57.6%), moderate and strong staining	213.6	RFS,DSS	diagnosis ∼ locoregional recurrence/distant metastasis, diagnosis ∼ breast cancer death	M
Kamai	2010	Japan	UC-UUT	108	Grade 1-3	Western blotting	49/108 (45.4%), >2.68	41.0	OS,DFS	NA,NA	M
Li	2010	China	Colorectal cancer	73	A-D	IHC	32/73 (43.8%), >1.27	NA	DSS	NA	KM
Siu	2010	China	Ovarian cancer	76	I-IV	IHC	30/76 (39.5%), NA	48.0	DFS	NA	M
Thariat	2012	France	Head and neck cancer	69	I-IV	Western blotting	NA, >0.47	38.0	DFS	diagnosis ∼ first relapse	M
Xu	2012	China	Hepatocellular carcinoma	52	I-IV	IHC	21/52 (40.4%), NA	NA	OS	NA ∼ death/last follow-up	M
Li	2013	China	Gastroesophageal junction adenocarcinoma	113	II-III	IHC	82/113 (72.6%), score>6	NA	OS	surgery ∼ NA	M
Han	2014	China	Pancreatic cancer	72	I-IV	IHC	38/72 (52.8%), score >=4	NA	OS	diagnosis ∼ death/last follow up	M
Qian	2014	China	Gastric cancer	131	I-IV	Agilent 244K array CGH platform	6/131 (4.6%), (logRatio>=0.8 & frequency>=5%) or (logRatio>2 & frequency>=2%)	NA	OS	NA	NA
Ong	2015	UK and Canada	Breast cancer	980	Grade I-III	Affymetrix SNP6.0 array	NA, >5 copies	150.0	OS	diagnosis ∼ NA	M
Park	2015	South Korea	Head and neck cancer	119	I-IV	IHC	50/119 (42.0%), score>=3	NA	OS,DSS	NA,NA	KM
Song	2015	China	Colorectal cancer	82	III-IV	IHC	62/82 (75.6%), score>3	NA	PFS	NA	KM
Zhu^b^	2015	China	Renal cell carcinoma	119	I-IV	IHC	NA, NA	NA	OS	surgery ∼ death/last follow-up	M

a Study investigated the prognostic effect of p-PAK1 only and was excluded from quantitative analysis.

b Study investigated the prognostic effect of p-PAK1 only and was excluded from quantitative analysis.

Abbreviations; UC-UUT, urothelial carcinoma of the upper urinary tract; NA, not available; IHC, immunohistochemistry; RFS, recurrence-free survival; OS, overall survival; PFS, progression-free survival; DSS, disease-specific survival; DFS, disease-free survival; M, multivariate cox proportional hazard regression; KM, Kaplan-Meier method.

### Meta-analysis

**Figure 2:**
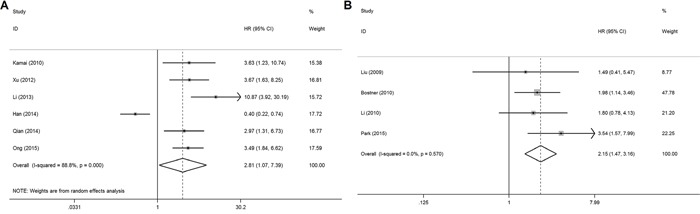
Forest plots of the meta-analysis of the association between PAK1 expression and the prognosis of patients with solid tumors **A.** Overall survival (using only individual HRs extracted directly from the original articles) **B.** Disease-specific survival. Abbreviations: HR, hazard ratio; CI, confidence interval.

**Figure 3:**
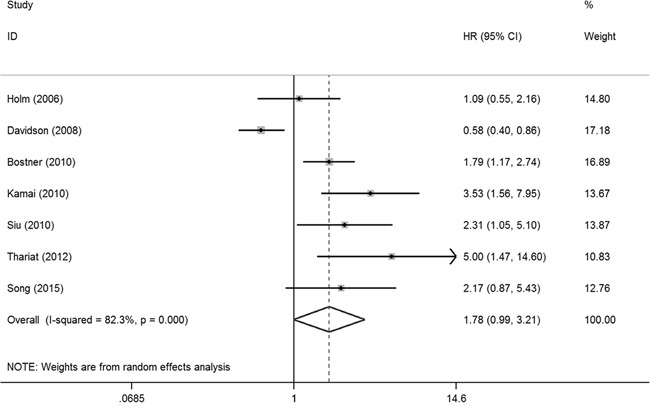
Forest plot of the meta-analysis of the association between PAK1 expression and solid tumor progression Abbreviations: HR: hazard ratio; CI: confidence interval.

In the meta-analysis, three outcome endpoints including DFS, PFS, and RFS that were similar in meaning were combined to use a unified prognostic parameter, time to tumor progression (TTP) instead. The meta-analysis of PAK1 expression was therefore based on three outcome endpoints: OS, DSS and TTP. Eight studies were included in the meta-analysis of OS. A random effects model was used to calculate the pooled hazard ratio (HR) and 95% confidence interval (CI) because the heterogeneity test reported a *P* value of less than 0.01. No significant association was observed between PAK1 expression and OS (pooled HR = 2.08, 95% CI = 0.93-4.64) (Supplementary Figure S1). Because some individual HRs were indirectly estimated (see Materials and Methods) and were therefore less reliable, we also performed a meta-analysis of OS using only the individual HRs extracted directly from the original articles. Six studies were included in that analysis, and again the heterogeneity test reported a *P* value of less than 0.01. We therefore used a random effects model to calculate the pooled HR and 95% CI. In this analysis, a significant relationship between PAK1 expression and OS among patients with solid tumors was detected (pooled HR = 2.81, 95% CI = 1.07-7.39) (Figure 2A). Four studies were included in the meta-analysis of DSS. A fixed effects model was used to calculate the pooled HR and 95% CI because the heterogeneity test reported a *P* value of 0.570. The result provided evidence of an association between PAK1 expression and DSS (pooled HR = 2.15, 95% CI = 1.47-3.16) (Figure 2B). Seven studies were used in the meta-analysis for TTP. The heterogeneity test reported a *P* value of less than 0.01, so a random effects model was used to calculate the pooled HR and 95% CI. No significant association between PAK1 expression and TTP was detected (pooled HR = 1.78, 95% CI = 0.99-3.21) (Figure 3). The results of our meta-analysis thus suggest that PAK1 expression may be a predictive marker of OS and DSS in patients with solid tumors, but it is not predictive of TTP.

### Publication bias test results

**Figure 4:**
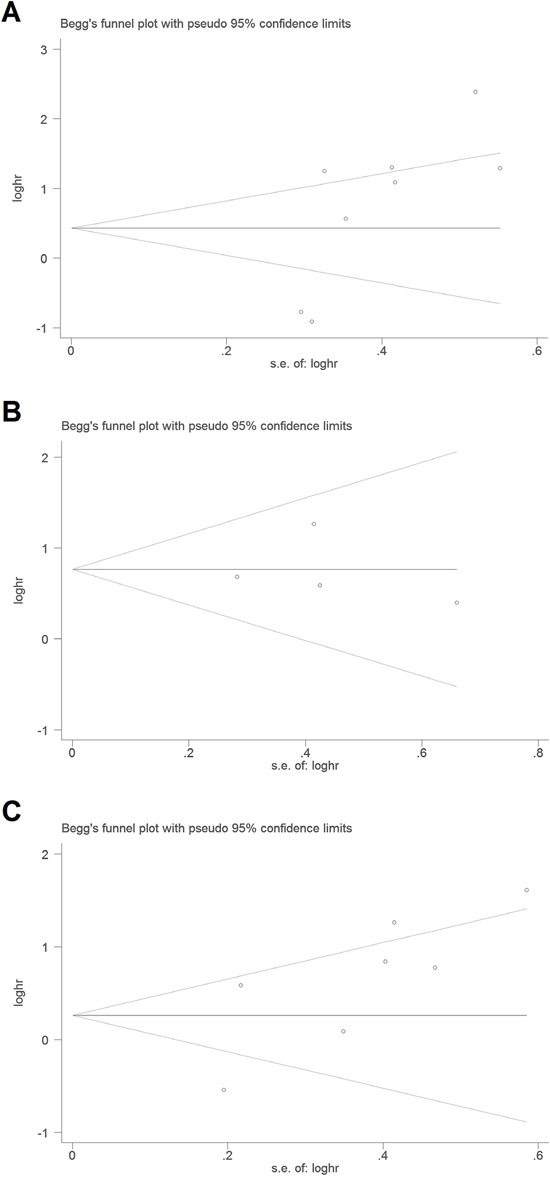
Begg's funnel plots for the studies involved in the meta-analysis of PAK1 expression and the prognosis of patients with solid tumors **A.** Overall survival. **B.** Disease-specific survival. **C.** Time to tumor progression. Abbreviations: loghr, logarithm of hazard ratios; s.e., standard error.

The Begg's funnel plot (Figure 4) and Egger's test showed there was no publication bias for DSS (*P* = 0.901) or for TTP (*P* = 0.062). However, publication bias may exist for OS (*P* = 0.032) in the analysis of high versus low PAK1 expression.

## DISCUSSION

The results of our meta-analysis suggest that higher tumoral PAK1 expression is associated with an unfavorable prognosis and is predictive factor associated with OS and DSS in patients with solid tumors. PAK1 is an effector of small Rho GTPases (Cdc42 and Rac1) [2]. PAK1 and Rac1 reportedly play important roles within cancer cell signaling networks and contribute to invasive and metastatic phenotypes [22, 23]. On the other hand, our meta-analysis indicates that PAK1 expression is not significantly associated with TTP in patients with solid tumors. The heterogeneity across the included studies is one potential reason for this. In addition, the combined effects of PAK1 with other molecular and environmental factors likely differ among cancer types.

Our meta-analysis has several limitations, so the results should be considered with a degree of caution. One limitation is that the sample size was not sufficient, particularly for the analysis of DSS. A second limitation is the heterogeneity caused by the diverse methods used to detect PAK1 expression and the varied cutoff values used in individual studies. The third limitation is that the patient data were not adjusted to account for details of the patients' characteristics, such as age and lifestyle. In addition, subgroup meta-analysis based on cancer type, PAK1 nuclear localization and p-PAK1 expression could not be carried out with the existing data. To achieve a more convincing conclusion, further analysis using a larger sample size, a unified detection method and adjusted individual data will be required, along with a stratified analysis based on cancer type, PAK1 nuclear localization and p-PAK1 expression.

## MATERIALS AND METHODS

### Literature search, selection and data collection

For this study, we searched for papers published before May 6, 2015 using the keywords “p21 protein (Cdc42/Rac)-activated kinase 1”/“PAK1”/“PAKalpha”, “cancer”/“tumor”/“neoplasm”/“carcinoma”, and “survival”/“prognosis”/“mortality”/“death” independently in PubMed and Web of Science. Among the papers identified, were further selected for the meta-analysis using the following selection criteria. 1) The full text of the study was in English. 2) The study provided adequate data for individual HRs and 95% CIs to be extracted or calculated [24]. 3) When studies sharing the same patient sample were compared, the most complete study among them was included in our meta-analysis.

Three investigators independently collected data from each eligible paper. The data collected included the name of first author, publication year, patients' country of origin, cancer type, number of patients, cancer stage or grade, detection method, percentage exhibiting high PAK1 expression and the corresponding cutoff value, median follow-up months, outcome endpoints, outcome definition, survival analysis method, and the HR and 95% CI for the high PAK1 expression group versus low PAK1 expression group. Individual HRs and 95% CIs were estimated [24] if only Kaplan-Meier survival plots were available. Multivariate HRs and 95% CIs were selected if both univariate and multivariate results were reported in an individual study. By checking among the three investigators, the final data collected was determined.

### Meta-analysis methods

Using the data collected from each eligible paper, we performed a meta-analysis of the outcomes to evaluate the relationship between PAK1 and solid cancer prognosis. Stata version 14.0 (Stata Corporation, College Station, TX, USA) was used to carry out the statistical analysis. Because the outcome endpoints DFS, PFS and RFS are similar in meaning, they were combined and a unified prognostic parameter, TTP, was used for the meta-analysis. Pooled HRs and 95% CIs for three outcome endpoints (OS, DSS, and TTP) were calculated. All the pooled HRs and 95% CIs were calculated using a fixed effects or random effects model. The model was chosen using a heterogeneity test. For the heterogeneity test, we performed the χ^2^-based *Q*-test [25]. When the *Q*-test reported a *P* value of more than 0.10, a fixed effects model was used to calculate the pooled HRs [26], otherwise random effects model was used [27].

Publication bias was tested using Begg's funnel plot and the Egger's test [28]. If the funnel plot was asymmetric and the Egger's test reported a *P* value of less than 0.05, publication bias was deemed to probably exist.

## SUPPLEMENTARY FIGURE



## References

[1] Dummler B, Ohshiro K, Kumar R, Field J (2009). Pak protein kinases and their role in cancer. Cancer Metastasis Rev.

[2] Narumiya S, Ishizaki T, Watanabe N (1997). Rho effectors and reorganization of actin cytoskeleton. FEBS Lett.

[3] Han J, Wang F, Yuan SQ, Guo Y, Zeng ZL, Li LR, Yang J, Wang DS, Liu MY, Zhao H, Liu KY, Liao JW, Zou QF (2014). Reduced expression of p21-activated protein kinase 1 correlates with poor histological differentiation in pancreatic cancer. BMC Cancer.

[4] Zhang S, Han J, Sells MA, Chernoff J, Knaus UG, Ulevitch RJ, Bokoch GM (1995). Rho family GTPases regulate p38 mitogen-activated protein kinase through the downstream mediator Pak1. J Biol Chem.

[5] Frost JA, Xu S, Hutchison MR, Marcus S, Cobb MH (1996). Actions of Rho family small G proteins and p21-activated protein kinases on mitogen-activated protein kinase family members. Mol Cell Biol.

[6] Holm C, Rayala S, Jirström K, Stål O, Kumar R, Landberg G (2006). Association between Pak1 expression and subcellular localization and tamoxifen resistance in breast cancer patients. J Natl Cancer Inst.

[7] Aoki H, Yokoyama T, Fujiwara K, Tari AM, Sawaya R, Suki D, Hess KR, Aldape KD, Kondo S, Kumar R, Kondo Y (2007). Phosphorylated Pak1 level in the cytoplasm correlates with shorter survival time in patients with glioblastoma. Clin Cancer Res.

[8] Davidson B, Shih IeM, Wang TL (2008). Different clinical roles for p21-activated kinase-1 in primary and recurrent ovarian carcinoma. Hum Pathol.

[9] Liu F, Li X, Wang C, Cai X, Du Z, Xu H, Li F (2009). Downregulation of p21-activated kinase-1 inhibits the growth of gastric cancer cells involving cyclin B1. Int J Cancer.

[10] Bostner J, Skoog L, Fornander T, Nordenskjöld B, Stål O (2010). Estrogen receptor-alpha phosphorylation at serine 305, nuclear p21-activated kinase 1 expression, and response to tamoxifen in postmenopausal breast cancer. Clin Cancer Res.

[11] Kamai T, Shirataki H, Nakanishi K, Furuya N, Kambara T, Abe H, Oyama T, Yoshida K (2010). Increased Rac1 activity and Pak1 overexpression are associated with lymphovascular invasion and lymph node metastasis of upper urinary tract cancer. BMC Cancer.

[12] Li LH, Zheng MH, Luo Q, Ye Q, Feng B, Lu AG, Wang ML, Chen XH, Su LP, Liu BY (2010). P21-activated protein kinase 1 induces colorectal cancer metastasis involving ERK activation and phosphorylation of FAK at Ser-910. Int J Oncol.

[13] Siu MK, Wong ES, Chan HY, Kong DS, Woo NW, Tam KF, Ngan HY, Chan QK, Chan DC, Chan KY, Cheung AN (2010). Differential expression and phosphorylation of Pak1 and Pak2 in ovarian cancer: effects on prognosis and cell invasion. Int J Cancer.

[14] Thariat J, Bensadoun RJ, Etienne-Grimaldi MC, Grall D, Penault-Llorca F, Dassonville O, Bertucci F, Cayre A, De Raucourt D, Geoffrois L, Finetti P, Giraud P, Racadot S (2012). Contrasted outcomes to gefitinib on tumoral IGF1R expression in head and neck cancer patients receiving postoperative chemoradiation (GORTEC trial 2004-02). Clin Cancer Res.

[15] Xu J, Liu H, Chen L, Wang S, Zhou L, Yun X, Sun L, Wen Y, Gu J (2012). Hepatitis B virus X protein confers resistance of hepatoma cells to anoikis by up-regulating and activating p21-activated kinase 1. Gastroenterology.

[16] Li Z, Zou X, Xie L, Dong H, Chen Y, Liu Q, Wu X, Zhou D, Tan D, Zhang H (2013). Prognostic importance and therapeutic implications of PAK1, a drugable protein kinase, in gastroesophageal junction adenocarcinoma. PLoS One.

[17] Qian Z, Zhu G, Tang L, Wang M, Zhang L, Fu J, Huang C, Fan S, Sun Y, Lv J, Dong H, Gao B, Su X (2014). Whole genome gene copy number profiling of gastric cancer identifies PAK1 and KRAS gene amplification as therapy targets. Genes Chromosomes Cancer.

[18] Ong CC, Gierke S, Pitt C, Sagolla M, Cheng CK, Zhou W, Jubb AM, Strickland L, Schmidt M, Duron SG, Campbell DA, Zheng W, Dehdashti S (2015). Small molecule inhibition of group I p21-activated kinases in breast cancer induces apoptosis and potentiates the activity of microtubule stabilizing agents. Breast Cancer Res.

[19] Park J, Kim JM, Park JK, Huang S, Kwak SY, Ryu KA, Kong G, Park J, Koo BS (2015). Association of p21-activated kinase-1 activity with aggressive tumor behavior and poor prognosis of head and neck cancer. Head Neck.

[20] Song B, Wang W, Zheng Y, Yang J, Xu Z (2015). P21-activated kinase 1 and 4 were associated with colorectal cancer metastasis and infiltration. J Surg Res.

[21] Zhu Y, Liu H, Xu L, An H, Liu W, Liu Y, Lin Z, Xu J (2015). p21-activated kinase 1 determines stem-like phenotype and sunitinib resistance via NF-κB/IL-6 activation in renal cell carcinoma. Cell Death Dis.

[22] Sahai E, Marshall CJ (2002). RHO-GTPases and cancer. Nat Rev Cancer.

[23] Kumar R, Gururaj AE, Barnes CJ (2006). p21-activated kinases in cancer. Nat Rev Cancer.

[24] Tierney JF, Stewart LA, Ghersi D, Burdett S, Sydes MR (2007). Practical methods for incorporating summary time-to-event data into meta-analysis. Trials.

[25] Lau J, Ioannidis JP, Schmid CH (1997). Quantitative synthesis in systematic reviews. Ann Intern Med.

[26] Mantel N, Haenszel W (1959). Statistical aspects of the analysis of data from retrospective studies of disease. J Natl Cancer Inst.

[27] DerSimonian R, Laird N (1986). Meta-analysis in clinical trials. Control Clin Trials.

[28] Egger M, Davey Smith G, Schneider M, Minder C (1997). Bias in meta-analysis detected by a simple, graphical test. BMJ.

