# Supplementary material for: p21-activated kinase 1 (PAK1) expression correlates with prognosis in solid tumors: A systematic review and meta-analysis
Source: Oncotarget. 2016 Mar 24;7(19):27422–9. doi: 10.18632/oncotarget.8320 (PMC5053660; doi:10.18632/oncotarget.8320)
Supplement: Supplementary file 1 [file oncotarget-07-27422-s001.pdf]

SUPPLEMENTARY FIGURE

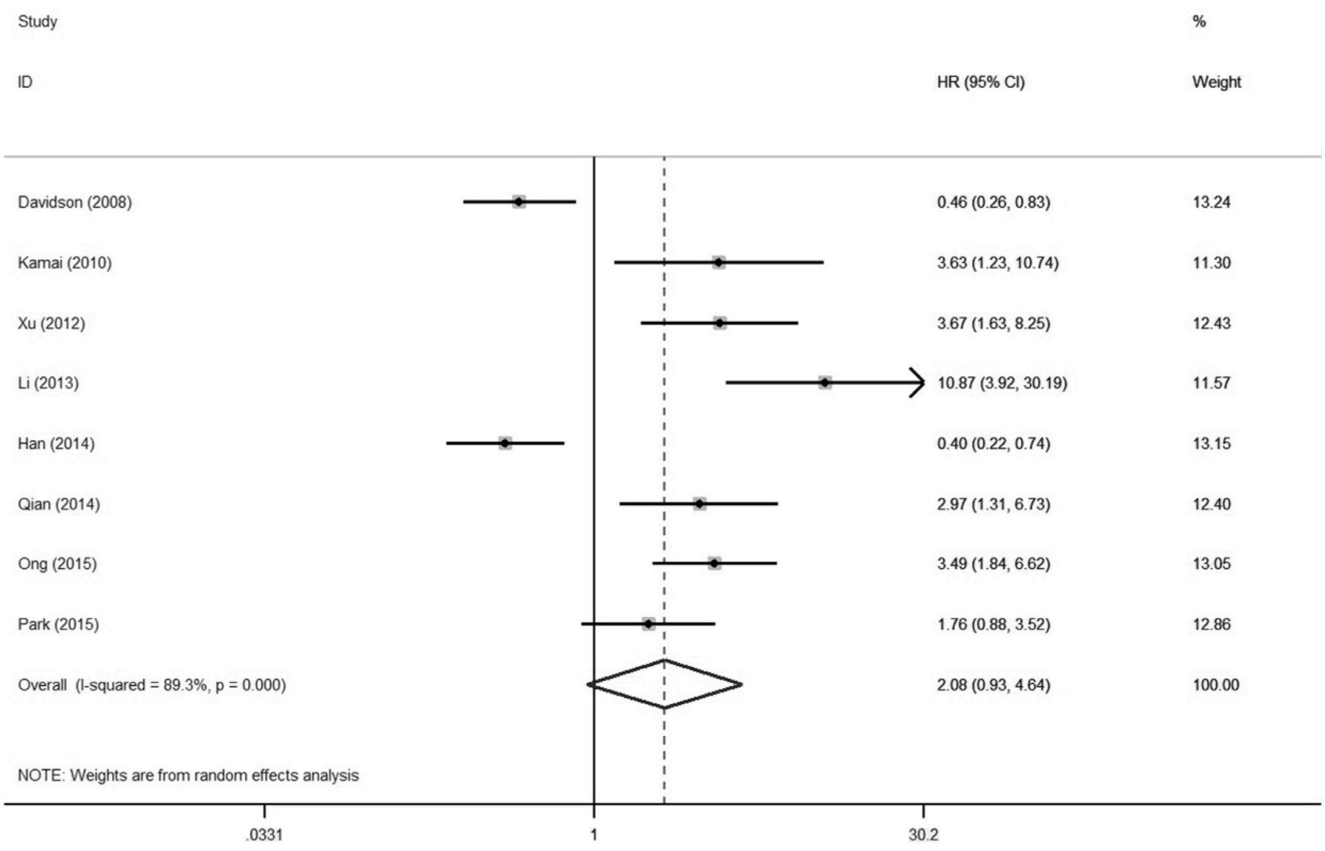

Supplementary Figure S1: Forest plot of the meta-analysis of the association between PAK1 expression and the overall survival of patients with solid tumors. Abbreviations: HR: hazard ratio; CI: confidence interval.
